# Factors Affecting Confirmed COVID-19 Patient's Recovery Time at King Abdulaziz Medical City, Jeddah

**DOI:** 10.7759/cureus.34130

**Published:** 2023-01-24

**Authors:** Majid S Al-Thaqafy, Rina Batahaf, Rinad Bahakeem, Rahaf Batarjee, Wejdan Mubaraki, Asim Alsaedi, Lamiaa A Alghamdi, Ziyad F Al Nufaiei

**Affiliations:** 1 Infection Prevention and Control, King Abdulaziz Medical City, Jeddah, SAU; 2 Epidemiology and Public Health, King Abdullah International Medical Research Center, Jeddah, SAU; 3 College of Applied Medical Sciences, King Saud Bin Abdulaziz University for Health Sciences, Jeddah, SAU; 4 Respiratory Therapy, King Saud Bin Abdulaziz University for Health Sciences, Jeddah, SAU; 5 Research Center, King Abdullah International Medical Research Center, Jeddah, SAU; 6 Infection Prevention and Control Department, King Abdulaziz Medical City, Jeddah, SAU; 7 Internal Medicine, King Abdullah International Medical Research Center, Jeddah, SAU; 8 College of Medicine, King Saud Bin Abdulaziz University for Health Sciences, Jeddah, SAU; 9 Infection Prevention and Control, King Abdulaziz Medical City/Ministry of National Guard Health Affairs, Jeddah, SAU; 10 Respiratory Care, King Saud Bin Abdulaziz University for Health Sciences, Jeddah, SAU

**Keywords:** copd: chronic obstructive pulmonary disease, spike (s) glycoprotein, angiotensin-converting enzymes 2, acute respiratory distress syndrome [ards], covid-19 outbreak, sars-cov-2 (severe acute respiratory syndrome coronavirus -2)

## Abstract

Introduction: The COVID-19 pandemic has become a threat to the public’s health, especially to the elderly and those with chronic conditions. It is capable of spreading from carriers who are both asymptomatic and symptomatic. Associated factors such as age, sex, severe symptoms of COVID-19 disease, and chronic disease have a significant impact on the recovery time of patients.

Aim: The study aimed to determine associated factors on recovery time in COVID-19 patients hospitalized at King Abdulaziz Medical city. Methods: A single-center retrospective study was utilized to recruit 1776 confirmed COVID-19 patients from 13 September to 24 October 2020 at King Abdulaziz Medical City (KAMC) in Jeddah.

Results: The patients were categorized into three age groups: below 5 years, 5 to 65 years, and above 65 years. The number of male patients in each group was 49, 764, and 73, and the number of female patients in each group was 54, 754, and 82, respectively. Impact recovery time on female patients was 11.75 days; with male patients was 10.95 days. Symptoms such as sore throat, diarrhea, and fever in female patients declined the recovery time. On the other hand, symptoms such as runny nose, diarrhea, fever, and headache in male patients declined the recovery time.

Discussion and Conclusion: It was revealed that older aged COVID-19 patients, male sex, and some symptoms decline recovery time. The study findings show an independent predictor of particular symptoms and sign which delay the time of recovery in the COVID-19 patients enrolled in the study differently, for male and female patients. Thus, patients who are infected with COVID-19 should be monitored keenly to prevent a prolonged rate of recovery and should be eligible for priority management to enhance a good clinical outcome.

## Introduction

In late December 2019, 27 reported cases of pneumonia of unrecognized etiology emerged from Wuhan city, Hubei province, which is located at the center of China [1]. Several epidemiological investigations had been made by the emergency response team sent by the Chinese Center for Disease Control (CCDC), and the initial result revealed that all 27 reported cases had a common exposure to Wuhan's Huanan Seafood Wholesale Market; therefore, closing down and disinfection order had become an obligation [2]. On 7 January 2020, throat swap samples were conducted from infected patients by CCDC to identify the causative agent named later by the International Committee on Taxonomy of viruses as severe acute respiratory syndrome Coronavirus-2 (SARS-CoV-2) [2,3]. On 11 February 2020, the World Health Organization (WHO) stated a final name for the pandemic disease caused by SARS- CoV-2: Coronavirus disease (COVID-19) [3].

As COVID-19 is a new epidemic, recent research has been published to clarify and identify the unknown virus from different aspects, including the virus’s nature. Laboratory confirmed that the new virus belongs to the coronavirus family, specifically to the genus beta coronavirus, since they have many standard features such as an envelope, spherical shape, single-strand RNA, and large-diameter ranges from 60 to 140 nm [4]. Moreover, the most suggested reservoir for SARS-CoV-2 is bats since their genome sequence analysis was identical 96.2% to the SARS-CoV-2 genome [5,6]. The genome of a novel virus encodes some essential proteins, such as spike (S) glycoprotein, the protein that is responsible for the attachment to angiotensin-converting enzymes 2 (ACE2) receptor allowing virus entry to the human body, and it is transmitted via respiratory droplets and direct contact [5,7]. According to studies, the recent coronavirus was detected in the gastrointestinal tract, saliva, and urine of confirmed patients from Wuhan and the United States, which suggests the probability of a fecal-oral transmission [5,8]. Confirmed patients mostly presented with mild manifestations such as fever, dry cough, sore throat, and dyspnea. However, some develop severe complications, including organ failure, pulmonary edema, severe pneumonia, septic shock, and acute respiratory distress syndrome (ARDS) [1]. In addition, psycho-sensorial syndrome, loss of smell and taste, has been identified in some asymptomatic patients; thus, The British Association of Otorhinolaryngology (ENT-UK) strongly recommended considering it as a significant symptom of COVID-19 infection [7].

Based on studies, comorbidities can be a severe risk factor for COVID-19 patients. A meta-analysis included six different studies with a total of 1527 confirmed patients and concluded that the most prevalent comorbidities were Hypertension 17.1%, Cardia-cerebrovascular diseases 16.4%, and Diabetes 9.7%. However, Cardia-cerebrovascular diseases were three folds higher in ICU severe cases [9]. Another study included 10 articles with a total of 76993 among infected patients, summarized that in addition to the previous comorbidities, smoking, Chronic Obstructive Pulmonary Disease (COPD), malignancy, and chronic kidney disease are considered the most prevalent diseases among COVID-19 patients [10]. Most published research confirmed that COVID-19 patients who are at an advanced age and have chronic comorbidities, including diabetes and hypertension, were at high risk of severe COVID-19 and mortality; however, no clear evidence proved the association between SARS-CoV-2 and persistent diseases mechanism [11-13].

According to published research that discussed the effect of certain comorbidities on COVID-19 patient outcomes and mortality rate, chronic comorbidities were confirmed to be significant contributing factors in the deterioration of COVID-19 patients' status. A study published on 24 March 2020 investigate the impact of hypertension on viral clearance. The findings showed that hypertension could contribute to delaying viral clearance [11,14]. However, there were not enough published studies globally and locally, especially in Saudi Arabia, explaining the relationship between having multiple comorbidities and recovery periods in COVID-19 patients. Also, there is a lack of studies in Saudi Arabia that reported the impact of different age groups and the severity of symptoms on COVID-19 patients’ recovery period [15,16].

Furthermore, respiratory therapists have a crucial role during the COVID-19 epidemic due to patients’ high demand for blood gas assessments, intubations, bronchoscopies, and mechanical ventilation. Since COVID-19 is an airborne disease, the Saudi Ministry of Health provided a special infection precaution guideline to minimize the risk of transmission among healthcare workers. As a result, respiratory therapists’ knowledge assessment is an important aspect that we must focus on [17].

Consequently, this study aims to determine the effect of comorbidity number, the severity of symptoms, and different age groups on confirmed COVID-19 patients’ recovery time and also to assess respiratory therapists’ knowledge regarding infection control precautions. Since our research is considered one of the few types of research that will be conducted in Saudi Arabia and discuss the current outbreak, the findings of this study will contribute to the local COVID-19 research in general and serve as a database at KAMC-Jeddah.

## Materials and methods

Study design and participants 

A single-center retrospective study was utilized to measure how the recovery time of COVID-19 patients hospitalized at King Abdulaziz Medical City is impacted by certain risk factors such as gender, communicable diseases and comorbidities number, signs and symptoms, hospital admission, and different age groups. The patients were recruited from 13 September to 24 October 2020 at King Abdulaziz Medical City (KAMC) in Jeddah. Data were collected from 1776 patients with confirmed COVID-19. Ethically, this study was approved by the Institutional Review Board of King Abdullah International Medical Research Center in Saudi Arabia (SP20/418/J).

Data collection

Real-time reverse-transcriptase polymerase chain reaction (RT-PCR) was utilized to confirm the diagnosis of COVID-19. Those patients who were diagnosed with COVID-19 were eligible to be included in the study. The patient's charts were retrospectively analyzed by utilizing the BESTCare system for the following factors: gender, communicable diseases and comorbidity number, signs and symptoms, hospital admission, and different age groups. The researcher loaded data into a Microsoft Excel (Redmond, USA) sheet. The data was secured for investigation only. 

Data analysis

IBM Corp. Released 2020. IBM SPSS Statistics for Windows, Version 27.0. Armonk, NY: IBM Corp was used to analyzing data. The data sheet loaded into a Microsoft Excel (Redmond, USA) sheet contains 16 variables. Those variables are segmented into three variable categories: Severity of symptoms, age group, and comorbidity number. The symptoms variables are fever, sore throat, cough, runny nose, shortness of breath, vomiting, diarrhea, fatigue, headache, nausea, level of consciousness, loss of taste/smell, and others. The age group was categorized into three levels: below 5 years, 5 to 65 years, and above 65 years. The objective of the study was to see if the patient’s recovery time was impacted by the categories listed above. In multiple regression, the R square value was used to measure how the independent variables are impacting dependent variables, hence, the recovery time. Furthermore, the regression model will identify which variable is significantly impacting the recovery time and what will be the difference in recovery time between those who had symptoms and who did not.

## Results

Characteristics of the participants

Some data have been excluded due to missing values. That lead us to include a total of 1776 patients who met the inclusion criteria of the study. Specifically, 886 patients were male, and 890 patients were female. The patients were categorized into three age groups: below 5 years, 5 to 65 years, and above 65 years. The number of male patients in each group was 49,764 and 73, respectively. The number of female patients in each group was 54,754 and 82, respectively.

Impact recovery time on female patients

The following multiple linear regression table shows an R Square value of 0.06. This explains that the independent variables, all together, impact the recovery time of a female patient by 6%. The p-value of the F statistic is less than 0.05. Therefore, the regression model can conclude that there is significant reliability at a 95% confidence interval. Among the female patients, cough and comorbidity numbers significantly impact recovery time, as the p-values of these two variables are less than 0.05. The minimum recovery time is 11.75 days. However, female patients with more than one symptom have less recovery time. These variables are fever with an intercept of -0.27, sore throat with an intercept of -0.43, SOB with an intercept of -0.10, and diarrhea with an intercept of -0.38. The age group also has a negative intercept of -0.58 (Table 1). As the patients get older, they are more capable of recovering from COVID-19. For instance, for patients belonging to group level 3 (above 65 years), the recovery time declined by 1.74 days. For the patient who had a fever, the recovery time declined by 0.27 days, the presence of a sore throat declined the time by 0.43 days, the presence of SOB declined the time by 0.10 days, and the presence of diarrhea caused recovery time by 0.38 days. All the other variables except will increase the recovery time, insignificantly, by their respective intercept values only if these are present in a patient.

**Table 1:** The Overall Significance of the Regression Model for Female and Male Patients

		Coefficients	Standard Error	t -Stat	P-value	Lower 95%	Upper 95%
Female	Intercept	11.75	0.91	12.86	0.00	9.95	13.54
	Fever >30	-0.27	0.37	-0.74	0.46	-0.99	0.45
	Sore throat	-0.43	0.42	-1.01	0.31	-1.26	0.40
	SOB	-0.10	0.43	-0.24	0.81	-0.94	0.74
	Diarrhea	-0.38	0.52	-0.73	0.47	-1.40	0.64
	Age Group	-0.58	0.44	-1.31	0.19	-1.44	0.29
Male	Intercept	10.95	1.01	10.81	0.00	8.96	12.94
	Sore throat	-0.33	0.50	-0.66	0.51	-1.30	0.65
	Runny Nose	-0.24	0.53	-0.45	0.65	-1.28	0.81
	Vomiting	-1.02	0.75	-1.36	0.18	-2.48	0.45
	Diarrhea	-0.29	0.59	-0.49	0.62	-1.46	0.87
	fatigue	-0.07	0.55	-0.13	0.89	-1.14	1.00
	Headache	-0.68	0.51	-1.34	0.18	-1.68	0.32
	Loss of taste/smell	-0.64	0.57	-1.13	0.26	-1.76	0.48
	Other	-0.39	0.31	-1.26	0.21	-1.01	0.22
	Age Group	-0.20	0.49	-0.40	0.69	-1.17	0.77
	Other	-0.39	0.31	-1.26	0.21	-1.01	0.22

Impact recovery time on male patients

Table 1, i.e., the multiple linear regression table, shows an R Square value of 0.08. This explains that the independent variables, all together, impact the recovery time of a male patient by 8%. The p-value of the F statistic is less than 0.05; therefore, the regression model can conclude that there is significant reliability at a 95% confidence interval. Among the male patients, cough, fever, nausea, and comorbidity number are significantly impacting on recovery time, as the p-values of these four variables are less than 0.05. The minimum recovery time is 10.95 days. However, male patients with more than one symptom have less recovery time. These variables are sore throat with an intercept of -0.33, runny nose with an intercept of -0.24, vomiting with an intercept of -1.02, diarrhea with an intercept of -0.29, fatigue with an intercept of -0.07, headache with an intercept of -0.68, loss of taste/smell with an intercept of -0.64 and others with an intercept of -0.39. The age group also has a negative intercept of -0.20. As the patients get older, they are more capable of recovering from COVID-19 (Table 1).

For instance, in patients belonging to group level 2 (5 to 65 years), the recovery time declined by 0.40 days. For those patients who had a runny nose, the recovery time declined by 0.24 days; the presence of a sore throat declined the time by 0.33 days; the presence of vomiting declined the time by 1.02 days, having diarrhea declined the time by 0.29 days, having fatigue declined the time by 0.07 days, and having headache declined the time by 0.68 days. In addition, a male patient who loses his taste/smell may recover earlier by 0.64 days compared to the patient who didn’t lose their taste. However, all the other variables showed an increase in the recovery time, insignificantly, by their respective intercept values, only if these are present in a patient. In fact, those male patients who got fever, cough, and nausea had a recovery time of 0.91, 1.91, and 1.60 days earlier, respectively, compared to a patient who did not. Finally, male patients who had more than one disease along with COVID-19 significantly had a higher recovery time.

## Discussion

Our study findings reported the minimum duration to recovery time from COVID-19 patients was 10.95 days in male patients with a mean age of 36.1±19.0 and 11.75 days in female patients with a mean age of 34.7±20.6. Recent findings from various other studies report 13 days in Ethiopia, 17 days in Hefei, China, 17 days in Vietnam, and 10 days in India [18-21]. However, the mean recovery time in both male and female patients was relatively lower than other reports of an average recovery time of COVID-19 patients from 18 days to 22 days and higher than reports of an average recovery time of 6.5 days [22-26]. The large difference in recovery days among the literature reported can be explained by the various healthcare services offered in these countries, Standard Operating Procedures (SOP) in place for COVID-19 patient management suitable for each country depending on the equipment and medication available for utilization, and the extent of disease severity in the country. These discrepancies in average recovery time also include study variations such as population structure, study setting, and study period. 

The present study found that older aged COVID-19 patients played an independent predictor of decreased recovery time in both the male and female patients recruited. For patients belonging to the age group of 65 years and above, the recovery time declined by 1.74 days. This can be argued as older patients take symptoms, and signs of COVID-19 infection more seriously compared to younger patients who generally take the symptoms and signs of COVID-19 infection. Thus, an early diagnosis and management can lead to a better prognosis [27]. On the contrary, multiple research findings report older aged patients to take a significantly longer time compared to younger aged patients [28-32]. The studies report that the longer recovery time may be because older age is accompanied by multiple comorbid conditions, which are a risk factor for the lower recovery rate and high morbidity and mortality. Old aged is also associated with decreased pulmonary function and immunity, which contribute greatly to poor recovery rates and clinical outcomes [28,29]. 

The study findings show an independent predictor of particular symptoms and sign which delay the time of recovery in the COVID-19 patients enrolled in the study. In the male patients, symptoms, and signs which delayed the recovery time were runny nose at 0.24 days, diarrhea at 0.29 days, sore throat at 0.33 days, and vomiting at 1.02 days. In comparison with the female patients, symptoms, and signs which delayed the recovery time were shortness of breath at 0.10 days, fever at 0.27 days, diarrhea at 0.38 days, and sore throat at 0.43 days. The symptoms which delayed recovery time in female patients were moderate COVID-19 compared to males, which were mild COVID-19 [27]. This can be explained in terms of sex-related differences that exist between males and females, as the latter is believed to have a stronger immune system compared to males and a higher level of cortisol and estrogen, which are beneficial [33]. 

The current study reported a significant association of comorbid conditions with an increased recovery time from the COVID-19 infection compared to non-comorbid conditions. Particular comorbid conditions such as diabetes mellitus, hypertension, liver disease, renal disease, and endocrinological problems are all independent predictors of an increased recovery time. All the comorbid conditions which show independent predictors affect the normal body physiology and function, which provide a platform for COVID-19 infection to progress in severity, causing an increased recovery rate [10,33-36]. Similar evidence support that comorbidities affect the recovery rate and prolong recovery time, particularly in old ages individuals in both developing and developed countries [37,38]. Patients with comorbidity conditions have a higher risk of delayed viral clearance.

Regression analysis conducted on symptoms and signs in relation to recovery time showed a significant association between a loss of taste and smell and early recovery. Loss of taste was a unique symptom associated with COVID-19 patients [39]. Studies report any prolonged recovery time associated with loss of taste and loss of smell linking it to the pandemic of long COVID [40,41]. Long COVID affects the majority of COVID-19 patients post-severe recovery, reporting that an estimated 80% of patients have one ongoing symptom at 60 days follow-up [42,43]. Most of the study patients had mild and moderate COVID-19 symptoms, which did not incline the study participants to have long COVID. 

Limitations

The study limitations are associated with the retrospective study design and the use of secondary data sources from patient files and hospital records. As a result, significant factors that would have influenced the duration of recovery of the patient from the COVID-19 infection were excluded. In addition, the study also had an unbalanced proportion of age categories which may have skewered the study findings. Clinical outcomes and incomplete baseline data were also excluded from the analysis, which would have provided insight into the variables that could influence the recovery rate from COVID-19 infection. 

## Conclusions

In general, this study found variables affecting prolonged recovery time from the COVID-19 infection in the patients hospitalized at King Abdulaziz Medical city. It was revealed that older aged COVID-19 patients, male sex, and some symptoms decline recovery time. The study findings show an independent predictor of particular symptoms and sign which delay the time of recovery in the COVID-19 patients enrolled in the study, different for male and female patients. Thus, patients who are infected with COVID-19 should be monitored keenly to prevent a prolonged rate of recovery and should be eligible for priority management to enhance a good clinical outcome. The study recommends that extensive follow-up should be conducted to monitor long COVID signs and symptoms. The long COVID is categorized as a long-term disease, which still plays a crucial role in determining recovery time.

## References

[1] Epidemiology Working Group for NCIP Epidemic Response (2020). The epidemiological characteristics of an outbreak of 2019 novel coronavirus diseases (COVID-19) in China. Zhonghua Liu Xing Bing Xue Za Zhi.

[2] Sohrabi C, Alsafi Z, O'Neill N (2020). World Health Organization declares global emergency: A review of the 2019 novel coronavirus (COVID-19). Int J Surg.

[3] Lai CC, Shih TP, Ko WC, Tang HJ, Hsueh PR (2020). Severe acute respiratory syndrome coronavirus 2 (SARS-CoV-2) and coronavirus disease-2019 (COVID-19): The epidemic and the challenges. Int J Antimicrob Agents.

[4] Wang C, Liu Z, Chen Z, Huang X, Xu M, He T, Zhang Z (2020). The establishment of reference sequence for SARS-CoV-2 and variation analysis. J Med Virol.

[5] Wu D, Wu T, Liu Q, Yang Z (2020). The SARS-CoV-2 outbreak: What we know. Int J Infect Dis.

[6] Wang L, Wang Y, Ye D, Liu Q (2020). Review of the 2019 novel coronavirus (SARS-CoV-2) based on current evidence. Int J Antimicrob Agents.

[7] Guo YR, Cao QD, Hong ZS (2020). The origin, transmission and clinical therapies on coronavirus disease 2019 (COVID-19) outbreak - an update on the status. Mil Med Res.

[8] Guan WJ, Ni ZY, Hu Y (2020). Clinical characteristics of coronavirus disease 2019 in China. N Engl J Med.

[9] Emami A, Javanmardi F, Pirbonyeh N, Akbari A (2020). Prevalence of underlying diseases in hospitalized patients with COVID-19: a systematic review and meta-analysis. Arch Acad Emerg Med.

[10] Sanyaolu A, Okorie C, Marinkovic A (2020). Comorbidity and its Impact on Patients with COVID-19. SN Compr Clin Med.

[11] Schiffrin EL, Flack JM, Ito S, Muntner P, Webb RC (2020). Hypertension and COVID-19. Am J Hypertens.

[12] Hussain A, Bhowmik B, do Vale Moreira NC (2020). COVID-19 and diabetes: Knowledge in progress. Diabetes Res Clin Pract.

[13] Bajgain KT, Badal S, Bajgain BB, Santana MJ (2021). Prevalence of comorbidities among individuals with COVID-19: A rapid review of current literature. Am J Infect Control.

[14] (2022). Hypertension and diabetes delay the viral clearance in COVID-19 patients. https://www.medrxiv.org/content/10.1101/2020.03.22.20040774v1.

[15] Shahid Z, Kalayanamitra R, McClafferty B (2020). COVID-19 and older adults: What we know. J Am Geriatr Soc.

[16] (2021). MOH Publications: COVID-19 guidelines. https://www.moh.gov.sa/en/Ministry/MediaCenter/Publications/Pages/covid19.aspx.

[17] Strickland SL, Roberts KJ, Smith BJ (2022). Burnout among respiratory therapists amid the COVID-19 pandemic. Respir Care.

[18] Kaso AW, Hareru HE, Kaso T, Agero G (2022). Time to recovery from Covid-19 and its associated factors among patients hospitalized to the treatment center in South Central Ethiopia. Environ Chall (Amst).

[19] Long Khuong Quynh, Hanh Hoang Hong, Hanh Tran Thi Tuyet, Quang La Ngoc, Van Minh Hoang (2020). Treatment for COVID-19 patients in Vietnam: Analysis of time-to-recovery. Pac J Trop Med.

[20] Guo A, Lu J, Tan H (2021). Risk factors on admission associated with hospital length of stay in patients with COVID-19: a retrospective cohort study. Sci Rep.

[21] Bhandari S, Tak A, Singhal S (2020). Patient flow dynamics in hospital systems during times of COVID-19: Cox proportional hazard regression analysis. Front Public Health.

[22] Alwafi H, Naser AY, Qanash S (2021). Predictors of length of hospital stay, mortality, and outcomes among hospitalised COVID-19 patients in Saudi Arabia: A cross-sectional study. J Multidiscip Healthc.

[23] Thiruvengadam G, Lakshmi M, Ramanujam R (2021). A study of factors affecting the length of hospital stay of COVID-19 patients by Cox-Proportional Hazard Model in a South Indian tertiary care hospital. J Prim Care Community Health.

[24] Tolossa T, Wakuma B, Seyoum Gebre D (2021). Time to recovery from COVID-19 and its predictors among patients admitted to treatment center of Wollega University Referral Hospital (WURH), Western Ethiopia: Survival analysis of retrospective cohort study. PLoS One.

[25] Zhou F, Yu T, Du R (2020). Clinical course and risk factors for mortality of adult inpatients with COVID-19 in Wuhan, China: a retrospective cohort study. Lancet.

[26] Liu X, Zhou H, Zhou Y (2020). Risk factors associated with disease severity and length of hospital stay in COVID-19 patients. J Infect.

[27] Chen YJ, Jian WH, Liang ZY (2021). Earlier diagnosis improves COVID-19 prognosis: a nationwide retrospective cohort analysis. Ann Transl Med.

[28] Hu X, Xing Y, Jia J (2020). Factors associated with negative conversion of viral RNA in patients hospitalized with COVID-19. Sci Total Environ.

[29] Xu J, Yang X, Yang L (2020). Clinical course and predictors of 60-day mortality in 239 critically ill patients with COVID-19: a multicenter retrospective study from Wuhan, China. Crit Care.

[30] Du RH, Liang LR, Yang CQ (2020). Predictors of mortality for patients with COVID-19 pneumonia caused by SARS-CoV-2: a prospective cohort study. Eur Respir J.

[31] Chen X, Zhu B, Hong W (2020). Associations of clinical characteristics and treatment regimens with the duration of viral RNA shedding in patients with COVID-19. Int J Infect Dis.

[32] Das AK, Gopalan SS (2020). Epidemiology of COVID-19 and predictors of recovery in the Republic of Korea. Pulm Med.

[33] Jin JM, Bai P, He W (2020). Gender differences in patients with COVID-19: Focus on severity and mortality. Front Public Health.

[34] Mehraeen E, Karimi A, Barzegary A (2020). Predictors of mortality in patients with COVID-19-a systematic review. Eur J Integr Med.

[35] Tadic M, Cuspidi C (2021). The influence of diabetes and hypertension on outcome in COVID-19 patients: Do we mix apples and oranges?. J Clin Hypertens (Greenwich).

[36] Boari GE, Chiarini G, Bonetti S (2020). Prognostic factors and predictors of outcome in patients with COVID-19 and related pneumonia: a retrospective cohort study. Biosci Rep.

[37] Ruan Q, Yang K, Wang W, Jiang L, Song J (2020). Clinical predictors of mortality due to COVID-19 based on an analysis of data of 150 patients from Wuhan, China. Intensive Care Med.

[38] Chinnadurai R, Ogedengbe O, Agarwal P (2020). Older age and frailty are the chief predictors of mortality in COVID-19 patients admitted to an acute medical unit in a secondary care setting- a cohort study. BMC Geriatr.

[39] Kye Wen Tan N, Jing-Wen C, Tan Tan (2022). The burden of prolonged smell and taste loss in covid-19. BMJ.

[40] Tan BK, Han R, Zhao JJ (2022). Prognosis and persistence of smell and taste dysfunction in patients with covid-19: meta-analysis with parametric cure modelling of recovery curves. BMJ.

[41] Ahmed H, Patel K, Greenwood DC (2020). Long-term clinical outcomes in survivors of severe acute respiratory syndrome and Middle East respiratory syndrome coronavirus outbreaks after hospitalisation or ICU admission: A systematic review and meta-analysis. J Rehabil Med.

[42] Connors JM, Levy JH (2020). COVID-19 and its implications for thrombosis and anticoagulation. Blood.

[43] Sigfrid L, Cevik M, Jesudason E (2021). What is the recovery rate and risk of long-term consequences following a diagnosis of COVID-19? A harmonised, global longitudinal observational study protocol. BMJ Open.

